# FeMn with Phases of a Degradable Ag Alloy for Residue-Free and Adapted Bioresorbability

**DOI:** 10.3390/jfb13040185

**Published:** 2022-10-13

**Authors:** Jan Tobias Krüger, Kay-Peter Hoyer, Jingyuan Huang, Viviane Filor, Rafael Hernan Mateus-Vargas, Hilke Oltmanns, Jessica Meißner, Guido Grundmeier, Mirko Schaper

**Affiliations:** 1 Materials Science, Paderborn University, Mersinweg 7, 33100 Paderborn, Germany; 2 DMRC-Direct Manufacturing Research Center, Paderborn University, Mersinweg 3, 33100 Paderborn, Germany; 3 Technical and Macromolecular Chemistry, Paderborn University, Warburger Str. 100, 33098 Paderborn, Germany; 4 Department of Veterinary Medicine, Institute of Pharmacology and Toxicology, Freie Universität Berlin, Koserstr. 20, 14195 Berlin, Germany; 5 Department of Pharmacology, Toxicology and Pharmacy, University of Veterinary Medicine Hannover, Foundation, Buenteweg 17, 30559 Hannover, Germany

**Keywords:** antibacterial behavior, biocompatibility, biomedical application, bioresorbable metal, corrosion, iron alloys, silver alloys

## Abstract

The development of bioresorbable materials for temporary implantation enables progress in medical technology. Iron (Fe)-based degradable materials are biocompatible and exhibit good mechanical properties, but their degradation rate is low. Aside from alloying with Manganese (Mn), the creation of phases with high electrochemical potential such as silver (Ag) phases to cause the anodic dissolution of FeMn is promising. However, to enable residue-free dissolution, the Ag needs to be modified. This concern is addressed, as FeMn modified with a degradable Ag-Calcium-Lanthanum (AgCaLa) alloy is investigated. The electrochemical properties and the degradation behavior are determined via a static immersion test. The local differences in electrochemical potential increase the degradation rate (low pH values), and the formation of gaps around the Ag phases (neutral pH values) demonstrates the benefit of the strategy. Nevertheless, the formation of corrosion-inhibiting layers avoids an increased degradation rate under a neutral pH value. The complete bioresorption of the material is possible since the phases of the degradable AgCaLa alloy dissolve after the FeMn matrix. Cell viability tests reveal biocompatibility, and the antibacterial activity of the degradation supernatant is observed. Thus, FeMn modified with degradable AgCaLa phases is promising as a bioresorbable material if corrosion-inhibiting layers can be diminished.

## 1. Introduction

Evermore diseases and injuries can be treated with implants to improve the quality of a patient’s life. Therefore, adapted materials to produce implants with suited properties for each application are necessary [1,2,3,4]. Most of the materials are developed by focusing on inert material behavior to guarantee biocompatibility, without considering the further potentially beneficial properties of materials [1,2,5,6]. Thus, the focus of actual research is to create implants with adapted stiffness, antibacterial properties, or the development of materials that enable bioresorption [3,7,8,9]. Bioresorbable implants degrade, while the remaining degradation products can be removed and excreted by the human body [1,4,7]. Accordingly, they are beneficial for applications where implants are only needed for a certain time such as a fixture of bone fracture in osteosynthesis. If these implants remain longer than necessary in the human body, they can lead to adverse consequences, and the removal is connected to patient burden and operational risk [3,7,8,9]. Some degradable materials, such as magnesium (Mg) alloys or polymers, are already used successfully. Nevertheless, they do not fulfill the requirements for all applications, such as the treatment of bone fracture or arteriosclerosis with stents. Thus, further materials such as molybdenum (Mo), zinc (Zn), and iron (Fe) are investigated [1,2,4,7,10,11,12]. Due to its mechanical properties and biocompatibility, Fe is a promising material [10,13,14,15,16]. Since Fe is a necessary trace element for the human organism, with a needed uptake of 8–27 mg per day corresponding to 9.8 g per year, the amount of Fe released by the dissolution of an osteosynthesis plate with a weight of 2 g over a few months is acceptable [17].

However, an adaption of pure Fe is necessary, since the degradation rate is too low, the mechanical properties are insufficient compared to commercially used Fe alloys, and the use of magnetic resonance imaging (MRI) is impossible. [3,10,12,18] Alloying with manganese (Mn) is a promising approach to overcome these limitations [13,14,19,20]. Mn stabilizes the paramagnetic austenitic structure, enabling MRI and improving the mechanical properties. High amounts of Mn evoke the effect of twinning-induced plasticity (TWIP), resulting in good strength and formability [12,21,22,23]. Moreover, a reduction in the electrochemical potential is obtained by the addition of Mn, causing an increased degradation rate compared to pure Fe [12,19,24]. Segregations of Mn result in local differences in the electrochemical potential and subsequently enhance the anodic dissolution, resulting in an overall increased degradation rate [12,16,20,24,25]. Shuai et al. [25], for instance, observed an approximately 2.5-times-higher degradation rate for Fe alloyed with 25 wt.-% Mn compared to pure Fe after a one-month static immersion test. However, the biocompatibility of Mn is critically discussed, and a dose of 4 mg per day corresponding to 1.5 g per year should not be exceeded [26,27]. The addition of carbon (C) reduces the Mn-amount necessary to achieve the TWIP-effect, causing enhanced biocompatibility [22,23]. Thus, steel containing Mn and C showing the TWIP-effect is selected for the present study.

Since, during degradation, the released ions react and form corrosion products, the consideration of the biocompatibility of single elements is not sufficient, and corrosion products generated in the complex environment of the living organism must be considered. Investigations of biocompatibility, even in vivo, consider the good biocompatibility of FeMn [13,19,27,28,29,30,31].

To fulfill the aim of bioresorbability, the degradation products must be excreted completely. Hardly dissolvable degradation products are observed at the implantation site of Fe-based stents, resulting in the brown discoloration of the tissue [29,32,33,34]. Since degradation products transported to the lymph nodes are observed 52 months after the implantation of Fe-stents, excretion by the lymphatic system is expectable. As a decomposition by macrophages is expectable as well, complete bioresorption should occur [16,32,35].

However, Dryndra et al. [28] reported biocompatibility but no significant degradation for in vivo-tested FeMn. For most applications, the degradation rate of FeMn is still too low, and an increase in the degradation rate is necessary. For the adaption of the degradation rate, the concentration of released ions during degradation has to be considered, as it is a decisive factor for biocompatibility [16,19,23,28,31]. The creation of phases with different electrochemical potentials to cause the anodic dissolution of less noble phases can increase the overall degradation rate. Thus, the addition of phases with high electrochemical potential to an FeMn matrix is aimed for. This strategy enables increased degradation, while the FeMn matrix with well-suited properties remains unchanged [22,36,37,38]. Noble elements with high electrochemical potential are promising in forming such phases. Due to the immiscibility of silver (Ag) with Fe in the liquid and solid states, Ag phases can exist inside an unchanged FeMn matrix. Additionally, Ag is biocompatible and has antibacterial properties [31,39,40,41]. Reports in the literature confirm an increased degradation rate due to the modification with Ag [22,31,35,39,42]. Dargush et al. [35] report an almost twice-as-large degradation rate after 12 weeks of in vivo degradation of Fe alloyed with 35 wt.-% Mn containing 1 wt.-% Ag compared to material without Ag. According to Huang et al. [39] the degradation rate of Fe with 5 wt.-% Ag is 50% higher than that for pure Fe after 30 days of immersion in Hank’s solution.

However, further aspects have to be considered to design such material. The various elements and chemical compounds in the human body result in the formation of various corrosion products, forming a multilayer structure [36,43,44]. These structures might affect further degradation. The released ions can form phosphates which deposit on the surface. Compounds with Fe and Mn are present on the surface, but the degradation can proceed further since exchange with the electrolyte is still possible [16,19,30,44]. In contrast, Ca-Phosphate (P), originating from the electrolyte, creates an inhibiting layer deteriorating the degradation rate [13,19,28,30,36,45]. Therefore, the formation of such inhibiting layers must be suppressed to enable further degradation. Wang et al. [44] suggest that noble phases can minimize the blocking effect, as a detachment of inhibiting layers and a changed structure can be observed. This suggestion is confirmed by the increased degradation rate (in vivo) of materials modified with Ag [35,38].

However, pure Ag is not suitable for modifying FeMn since the Ag particles would remain after the degradation of the matrix. This is not acceptable, as impairments may occur due to the remaining particles, and this contradicts the avoidance of such risks by complete bioresorption [46]. As particles can be transported through the human body, Ag might reach areas such as the brain, where the release of Ag is critically discussed [47,48,49]. Furthermore, impairments due to tissue damage by sharp edges, thrombosis, or strokes are possible [50,51]. The replacement of pure Ag with a completely degradable Ag alloy is an approach to prevent such consequences. Such alloy enables the degradation of Ag phases after the matrix, while their electrochemical potential is high enough to enhance the anodic dissolution of the FeMn matrix [41,52,53]. To the best of the authors’ knowledge, this study is the first to report on the modification of FeMn with a degradable Ag alloy, enabling a residue-free dissolution. Thus, the concern of remaining Ag particles after the degradation of the Fe-based matrix is addressed.

For the adaption of degradable Ag phases, it has to be considered that the biocompatibility of Ag depends on the concentration of released Ag ions and the total released amount of Ag [40,54,55]. If the concentration of Ag is too high, the mechanism responsible for the antibacterial effect harms the human cell, but a suitable concentration of Ag enables antibacterial properties without impairment of the organism [56,57,58]. Since implant-related infections are a major challenge for the use of implants, the simultaneous addressing of this issue is one major benefit of the addition of Ag [1,58,59]. The adjustment of the Ag release through alloying is therefore purposeful. A degradable Ag alloy containing calcium (Ca) and lanthanum (La) (AgCaLa) developed by Krüger et al. [41] is proven to be suitable for modifying FeMn. Ca is a necessary trace element, and La is successfully investigated as an alloying element of degradable Mg alloys, although the uptake should be strictly limited [60,61].

The fabrication of immiscible material combinations such as Fe and Ag is challenging. Powder-based processes such as the additive manufacturing method laser beam melting (LBM) enable the processing of such materials since powders can be mixed mechanically. During the subsequent continuous melting process characterized by a small melt pool, a strong melt flow, and rapid melting and solidification, the Ag is incorporated into the bulk material. By the modification of the raw material and the LBM process, the chemical composition and the morphology of the Ag and AgCaLa phases are adaptable [22,62,63,64]. The raw material for the AgCaLa phases is obtained by conventional alloying and subsequent gas atomization, whereas the final structure and chemical composition of the AgCaLa inside the FeMn matrix are influenced by the LBM process [41,64].

For the specific adjustment of the degradation behavior, the varying conditions in the human organism and the individual response of the human organism have to be considered. Achieving a reliable and suited degradation under all possible conditions is challenging, since the degradation of Fe-based alloys strongly depends on the ambient conditions. The degradation is affected by the ingredients of the solution, the pH value, which determines the solubility of the corrosion products, and the amount of dissolved gases, such as oxygen (O), that is required for the cathodic reaction [14,33,35,37,44]. For implant design, the slowest degradation must guarantee a sufficient supporting effect for healing, while the fastest degradation must enable the dissolution in a defined period.

However, the increased degradation of the FeMn matrix, the degradation of the AgCaLa particles after the matrix, biocompatibility, and an antibacterial effect are expectable but not guaranteed for FeMn modified with degradable AgCaLa. Therefore, an in-depth investigation of the listed properties is necessary, and the corresponding results are presented in this study. These results enable the further targeted adaption of the material to fulfill all requirements.

## 2. Materials and Methods

The investigated samples are produced via LBM using a mixture of FeMn (nominal composition: 22 wt.-% Mn, 0.6 wt.-% C and Fe bal.; Nanoval GmbH & Co. KG, Berlin, Germany) powder with 5 wt.-% AgCaLa (nominal composition: 12 wt.-% Ca, 5 wt.-% La and Ag bal.; Nanoval GmbH & Co. KG, Berlin, Germany) powder. As reference, pure FeMn and FeMn modified with 5 wt.-% pure Ag (ECKART TLS, Bitterfeld-Wolfen, Germany) are investigated. An amount of 5 wt.-% is selected, as the results of Huang et al. [39], Niendorf et al. [22], and Liu et al. [65] demonstrate enhanced degradation, biocompatibility, and acceptable mechanical properties for such an amount of Ag. The powders with a nominal particle size between 20 and 63 µm are produced via argon gas atomization. All powders have a suitable particle size distribution that is almost within the desired range and an almost spherical shape with only a few satellites [63,64]. Upright plates with a size of 2.5 × 10 × 12.5 mm³ are manufactured with an SLM 280^HL^ (SLM Solutions Group AG, Lübeck, Germany) in an argon atmosphere (layer thickness: 50 µm, scanning velocity: 750 mm/s, laser power: 280 W, hatch distance: 110 µm) [63,64]. The resulting FeMn matrix is characterized by an austenitic structure and segregations of Mn along with the melt-pool structure of LBM as well as the columnar dendritic solidification structure. The Ag and AgCaLa phases are homogeneously dispersed in the FeMn matrix and have a comparable morphology (approx. size: 30–370 µm). The Ag phases contain 88.3 wt.-% Ag, 11.7 wt.-% Mn, and 0.1 wt.-% O. Small slag particles are included in the AgCaLa phases. The AgCaLa phases contain 75.8 wt.-% Ag, 10.6 wt.-% Ca, 5.4 wt.-% La, 5.2 wt.-% Mn, and 3.9 wt.-% O. A detailed characterization of the LBM-processed materials and conventionally processed AgCaLa alloy has been carried out by Krüger et al. [41,63,64]. In the following, the samples are designated as FeMn, FeMnAg, and FeMnAgCaLa.

### 2.1. Electrochemical Investigations

A Potentiostat MLab200 (Bank Elektronik—Intelligent Controls GmbH, Pohlheim, Germany; Potential resolution: +/− 1 mV; Current resolution: 200 pA) with a three-electrode arrangement is used to determine the open circuit potential (OCP) and the linear sweep voltammetry (LSV). The reference electrode is an Ag/AgCl electrode, type SE11 in saturated KCl solution (Xylem Analytics Germany Sales GmbH & Co. KG, Weilheim, Germany), and the counter electrode is a platinum sheet. The samples are fixed in an inert sample holder with a circular contact surface of 24.4 mm². Ringer–Lactate solution (RL) (B. Braun SE, Melsungen, Germany) is used as the electrolyte. The samples are ground before the measurement with a grid size of P1200 to remove oxide layers. A stabilized state is achieved by waiting 5 min after arranging the set-up. During the OCP measurement, the data are recorded at 10 Hz for 10 min. The LSVs are collected with new samples between −1800 mV vs SHE and 1200 mV vs SHE, with a scan rate of 1 mV/s.

### 2.2. Scanning Kelvin Probe Force Microscopy (SKPFM)

SKPFM studies were performed to determine the contact potential differences between the matrix and the Ag phases. Samples were ground with SiC paper (grit P600, P1000, P2500, and P4000) and polished with 1 µm diamond paste (Schmitz-Metallographie GmbH, Herzogenrath, Germany). The polished samples were cleaned by sonication in ethanol for 10 min (Ultrasonic Cleaner, 45 kHz, 120 W, VWR International GmbH, Darmstadt, Germany). SKPFM measurements were performed using an MFP-3D-AFM setup (Oxford Instruments Asylum Research Inc., Santa Barbara, USA) with a platinum-coated tip (MikroMasch HQ:NSC15/Pt, 325 kH, 40 N m^−1^, NanoAndMore GmbH, Wetzlar, Germany). The delta height was set to 40 nm. The scan rate was 0.1 Hz, and the amplitude was 1 V. A 3 V bias voltage was applied to the probe during the measurement. In this case, a higher potential difference indicates a more negative electrode potential [66]. Three parallel samples of each alloy were measured.

### 2.3. Immersion Tests

The immersion tests are performed for 56 days under varying conditions (Table 1). Five different electrolytes (Table 2) are used to investigate the influence of the specific components of the immersion solution and the pH value: Hanks’ Solution (HS) with Ca++ and Mg++ (Lonza Group AG, Basel, Switzerland), RL, NaCl (NC, 0.9 %), pH3-, and pH5-solution. The solutions should imitate the conditions in the human body. For each combination, three samples are tested together in a laboratory glass bottle. One of three samples is ground iteratively up to P4000 before the immersion test to enable detailed investigations after immersion. The others are both used as build conditions. The samples have a size of 2.5 × 10 × 12.5 mm³, and a ratio of 9.2 mL/cm² is used for immersion. Holes with a diameter of 1 mm in the samples enable the free-hanging positioning in the immersion solution with a polypropylene filament. The influence of solved gases is investigated by the application of additional bubbling air (0.2 l/h), increasing the exchange with the atmosphere compared to aeration. Furthermore, the influence of temperature is investigated at 20 °C and 37 °C.

The samples are inspected after 1, 3, 6, and 10 days and, subsequently, every 7 days. At each inspection, the samples are removed from the solution, and the solution is exchanged. After the removal, the samples are rinsed (slow movement in a bath) in Ringer rinsing solution (B. Braun SE, Melsungen, Germany) and acetone. The samples are dried with hot air for 30 s. This treatment removes loose degradation products which imitate the conditions in the human body, where a complete removal does not occur but rather some flow and exchange of the surrounding solution. The sample weight is measured with an XP205 scale (Mettler-Toledo LLC, Columbus, OH, USA) with an accuracy of 0.01 g, and images of the samples are taken. After the immersion test, the samples are investigated via Raman spectroscopy and field emission scanning electron microscopy (FE-SEM). Therefore, a Zeiss Ultra Plus (Carl Zeiss AG, Oberkochen, Germany) with a secondary electron (SE) detector is used. The energy-dispersive X-ray spectroscopy (EDS) detector Octane Pro (AMETEK, Berwyn, PA, USA) of the SEM enables the determination of the chemical composition. Before investigating via SEM, the samples are cleaned for 30 s in an acetone ultrasonic bath.

The degradation behavior of the AgCaLa phases after release from the FeMn matrix is investigated with an immersion test over 6 months to enable the dissolution of the matrix and the release of Ag and AgCaLa particles, respectively. Half of the RL is changed every two weeks in order to guarantee the remaining released particles in the immersion vessels. To dissolve depositions after the immersion test, ultrasonic is applied for 30 s. The solution is removed from the immersion vessel, and the residues and samples are dried and investigated via SEM.

### 2.4. Raman Spectroscopy

Raman spectra were measured to identify corrosion products by using an InVia Renishaw Raman microscope (Renishaw plc, Wotton-under-Edge, UK) with a (charge-coupled device) CCD detector. A 532 nm yttrium-aluminum-garnet (YAG) laser, an 1800-L/mm grating and a 50× objective were used. The laser power at the sample was adjusted to 0.1 mW (power densities: 0.2 mW/µm^2^). The energy resolution was 0.3 cm^−1^ (full width at half maximum, FWHM).

### 2.5. Cytocompatibility Tests and Microbiological Analyses

The experiments are carried out with the murine fibroblast cell line L-929 (CLS Cell Lines Service GmbH, Eppelheim, Germany) and the human osteosarcoma cell line 87070202 (HOS; European Collection of Authenticated Cell Cultures Merck GmbH, Darmstadt, Germany). Fibroblasts and osteosarcoma cells were chosen since implanted alloys may come in contact with these cell types. Furthermore, L929 cells are recommended according to DIN ISO 10933-5 for viability testing. L-929 cells are grown and passaged in Roswell Park Memorial Institute (RPMI)-1640 medium (Biochrom GmbH, Berlin, Germany) supplemented with 10% fetal calf serum (FCS) superior (Biochrom GmbH, Berlin, Germany) and 1% penicillin/streptomycin (Pen/Strep) (Biochrom GmbH, Berlin, Germany). Eagle’s Minimum Essential Medium (EMEM)/Hanks’ (Carl Roth GmbH + Co. KG, Karlsruhe, Germany) maintained 10% FCS, 1% Pen/Strep, and 1% non-essential amino acids (Biochrom GmbH, Berlin, Germany), and 2 mM L-glutamine (Biochrom GmbH, Berlin, Germany) was used for HOS cells. Both cell lines are grown and passaged in cell culture flasks or multi-well plates (Greiner Bio-One GmbH, Frickenhaus Germany). A 0.05% trypsin/0.02% ethylene-diamine-tetraacetic acid solution (Biochrom GmbH, Berlin, Germany) is used for passaging. The cells are plated with a density of 10,000 cells per well in 96-well microtiter plates. For experimental exposure, each alloy is immersed in 10 mL of the cell culture medium or Mueller–Hinton broth (Carl Roth GmbH + Co. KG, Karlsruhe, Germany) without additives. After a 3-day incubation at 37 °C in a humidified atmosphere of 5% CO_2_, the cells undergo 24 h of incubation with the degradation medium in a humidified atmosphere at 37 °C and 5% CO_2_.

### 2.6. Cell Viability Test

Cell viability is determined by a 3-(4,5-dimethylthiazol-2-yl)-5-(3-carboxymethoxyphenyl)-2-(4-sulfophenyl)-2H-tetrazolium inner salt (MTS) assay (CellTiter 96^®^ Aqueous One Solution Cell Proliferation Assay, Promega Corp., Madison, WI, USA) with confluent cells after incubating with degradation media for 24 h, as previously described by Krüger et al. [41]. Each concentration is measured eight times, while each experiment is performed six times.

### 2.7. Test for the Antibacterial Activity of the Degradation Supernatant

Standard strains of *Escherichia coli* (*E. coli*; ATCC^®^ 25922, Leibniz Institute DSMZ-German Collection of Microorganisms and Cell Cultures GmbH, Braunschweig, Germany) are used since these bacteria are common etiologic agents of clinical infections involved in bone tissue infections related to implants. The frozen stock is incubated at 37 °C for 24 h on sheep blood agar plates (Thermo Fisher Scientific Inc., Waltham, MA, USA) before starting the experiments.

The in vitro exposure of *E. coli* to degradation media (as described above) is performed using 96-well microtiter plates with 10^4^ colony-forming units (cfu) per well. Each 96-well microtiter plate contains a set of six aliquots of every sample containing bacteria. After 24 h of incubation at 36 °C ± 1 °C, the optical density (OD) of each well of a microtiter plate is measured with a photometer at 450 nm. Each experiment is performed four times.

## 3. Results

**Figure 1:**
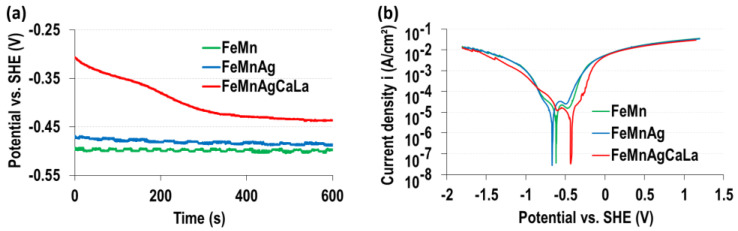
Electrochemical properties determined in RL: (**a**) transient of free corrosion potential; (**b**) linear sweep voltammetry.

The electrochemical analysis of the corrosion current densities as a function of the applied electrode potential (LSV curves) in RL is illustrated in Figure 1, along with the transient of the free corrosion potential open circuit potential (OCP) over the immersion time. The measured data indicate a change in the corrosion kinetics as a function of the alloy composition. The LSV data of FeMn and FeMnAg did not show any significant difference in the anodic and cathodic regions (Figure 1). In agreement with this result, the corresponding OCP data of FeMn and FeMnAg showed a similar trend.

The initial OCP values during the first 10 min of immersion (Figure 1a) show a more anodic corrosion potential for the FeMnAgCaLa alloy in comparison to the FeMn and FeMnAg alloy substrates. This result corresponded well with the corresponding LSV data showing that that the anodic dissolution rate is reduced for the FeMnAgCaLa alloy in comparison to the two other alloys.

At high anodic overpotentials, the current densities of all three alloys become similar, which can be explained by the dominating anodic dissolution of the FeMn phase in all cases.

**Figure 2:**
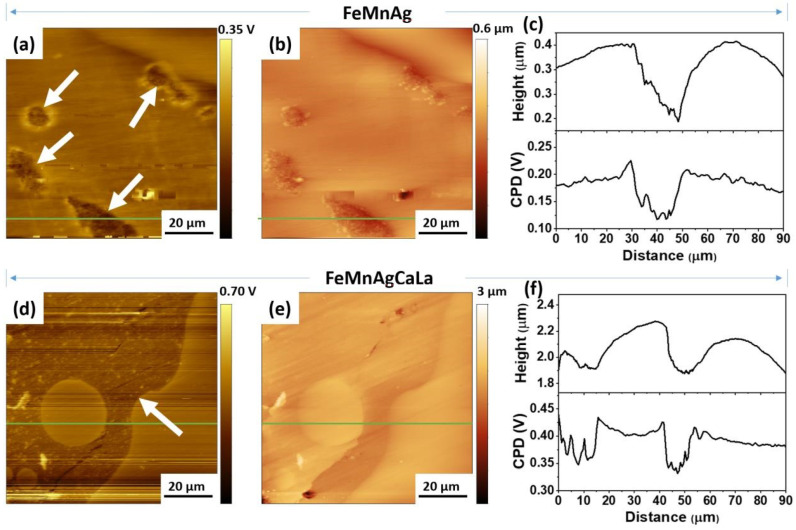
SKPFM results of FeMnAg and FeMnAgCaLa obtained from a 90 μm × 90 μm area: (**a**,**d**) topography, (**b**,**e**) corresponding contact potential differences, (**c**,**f**) corresponding cross-section (green line) profiles.

Figure 2 shows the AFM topographic and volta potential maps of polished FeMnAg (Figure 2a–c) and FeMnAgCaLa (Figure 2d–f). The SKPFM data clearly show a potential difference between the two main phases of FeMnAg (Figure 2a) and FeMnAgCaLa (Figure 2d).

The Ag and AgCaLa phases (white arrows) are darker, indicating a lower CPD value of Ag phases, which confirms the more noble character compared to the FeMn phase. The cross-section profiles (green line in Figure 2) determined by SKPFM indicate a difference of about 70 mV between the CPD values of the Ag and AgCaLa phases and the FeMn phase. In the same interval, the height profile determined by AFM shows that the Ag and AgCaLa phases form grooves on the surface after the polishing step due to their lower hardness. In addition, FeMnAgCaLa obtained a higher root mean square (rms) roughness value of 129.3 ± 49.2 nm compared to that of FeMnAg (48.8 ± 17.5 nm).

**Figure 3:**
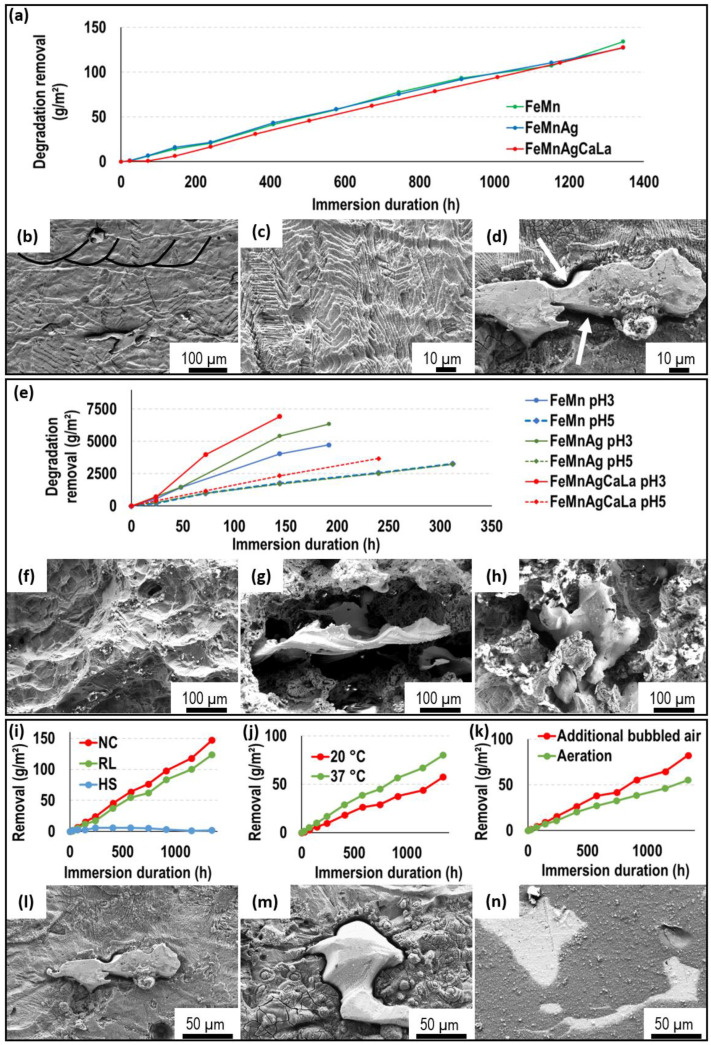
(**a**) Progress of degradation depending on material (N = 3, 37 °C, RL, aeration), SEM-SE: (**b**) FeMnAg after 700 h, (**c**) FeMn after 700 h, (**d**) FeMnAg after 1344 h; (**e**) progress of degradation depending on pH value (N = 3, 37 °C, aeration), SEM-SE: (**f**) FeMn after 192 h at pH3, (**g**) FeMnAg after 192 h at pH3; (**h**) FeMnAgCaLa after 144 h at pH3; progress of degradation of FeMnAg depending on (**i**) immersion solution (N = 12), (**j**) temperature (N = 18), (**k**) exchange with atmosphere (N = 18), SEM-SE after 1344 h: (**l**) FeMnAg (37 °C, NC, aeration), (**m**) FeMnAg (37 °C, RL, aeration), (**n**) FeMnAg (37 °C, HS, aeration).

During the immersion tests, the course of mass removal is similar for all three investigated materials under comparable conditions (Figure 3a). Thus, the expected increased degradation due to the addition of Ag and AgCaLa does not occur. The same results are obtained for all combinations of immersion conditions. The observation of the sample surface confirms the absence of a significant effect of the Ag and AgCaLa phases, as no crucial impact of the Ag phases on the FeMn is observed (Figure 3b,d). Just a small gap with a dimension of 1–3 µm around the Ag and AgCaLa phases has emerged (Figure 3d, white arrows). Such gaps have not been present before degradation [64]. The Ag and AgCaLa phases remain, while the matrix is dissolved, as expected. The structure of the melt pools (Figure 3b) and the structure of columnar dendritic solidification (Figure 3c) are observable. Thus, the structure of segregations of Mn along the microstructure observed by Krüger et al. [63] becomes visible during the immersion test, and the influence of segregations on the degradation is conclusive.

The degradation proceeds continuously. For long-time immersion tests over 6 months, continuous degradation is observed. A few more depositions are observed after 1344 h (Figure 3d) compared to 700 h (Figure 3c). These depositions do not significantly affect the degradation since mass removal proceeds continuously. Thus, a continuous transfer of function from the implant back to the human organism might be possible.

The pH value influences the degradation process significantly, as the removal is approximately 100 times higher than that for neutral pH values (Figure 3e). The removal at pH3 is higher than that at pH5. An increased degradation rate of FeMnAgCaLa compared to FeMn is observed at pH3 and pH5. An increased degradation due to the addition of pure Ag is observed at pH3. The course of mass removal is conclusive with the samples after immersion (Figure 3f–h), since the significantly increased removal of the matrix around the AgCaLa phase is observed. Thus, the expected increased degradation by modification with Ag and AgCaLa occurs for low pH values. As expected, the Ag and AgCaLa phases remain while the matrix degrades. This, together with the increased degradation of the matrix, indicates a higher corrosion resistance of Ag and AgCaLa and the effectiveness of a higher electrochemical potential for low pH values.

Aside from the pH value, the composition of the immersion solution, temperature, and intensity of exchange with the atmosphere affect the degradation rate. The curves in Figure 3i–k are the average material removal (FeMnAg) during immersion under varying conditions. As expected, higher temperatures and additional bubbling air increase the degradation rate (Figure 3j,k). Bubbling air intensifies the exchange with the air, enhancing the uptake of necessary O, and higher temperatures promote the chemical reactions. As for immersion in HS (Figure 3i), the weight is almost constant compared to immersion in RL and NC, and the components of the immersion solution have a significant influence. The constant weight during immersion in HS is conclusive since only slight depositions are observed on an almost unchanged sample surface (Figure 3n), whereas the sample surfaces after immersion in RL and NC are characterized by the removal of matrix material and depositions (Figure 3l,m). These depositions are more pronounced for immersion in RL, which might be correlated with the slightly lower mass removal compared to immersion in NC.

**Figure 4:**
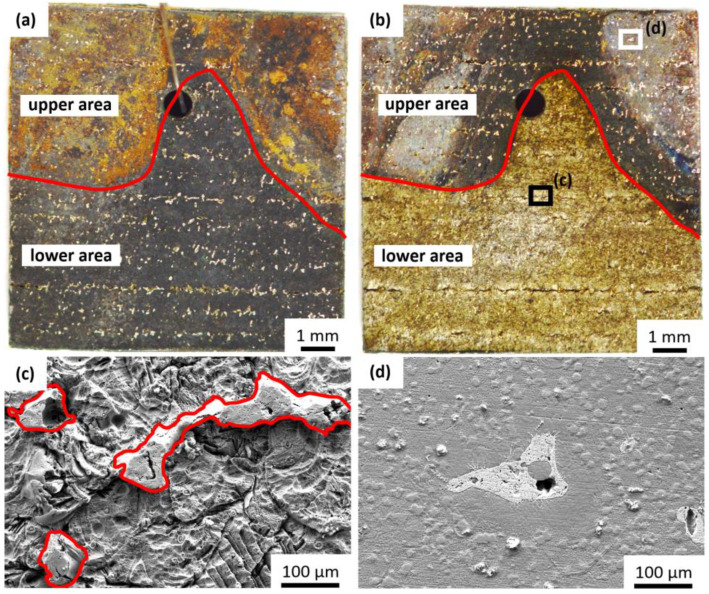
Local differences in degradation progress, FeMnAgCaLa after 1344 h (37 °C, RL, aeration): (**a**) image before ultrasonic cleaning; (**b**) image after ultrasonic cleaning; (**c**) SEM-SE: Magnified section of black box in (**b**); (**d**) SEM-SE: Magnified section of white box in (**b**).

The degradation proceeds continuously over time but heterogeneously over the sample surface. The FeMnAgCaLa sample shown in Figure 4 is covered by brown depositions in the upper corners after immersion. The rest, except for the AgCaLa phases, is covered by a black deposition (Figure 4a). These depositions are removable by ultrasonic cleaning (Figure 4b). The surface is characterized by the significant corrosion removal of the FeMn matrix in the lower area (Figure 4c) and slight corrosion in the upper corners (Figure 4d). The brown depositions might influence further degradation.

The AgCaLa phases (Figure 4c) are characterized by a more fragmented structure with pores compared to the Ag phases (Figure 3d). This structure is in accordance with the structure of AgCaLa phases before degradation, since some porosity in connection to the AgCaLa phases as well as inclusions of slag are observed. The dissolution of these inclusions is expectable and results in finely structured porosity [64].

**Figure 5:**
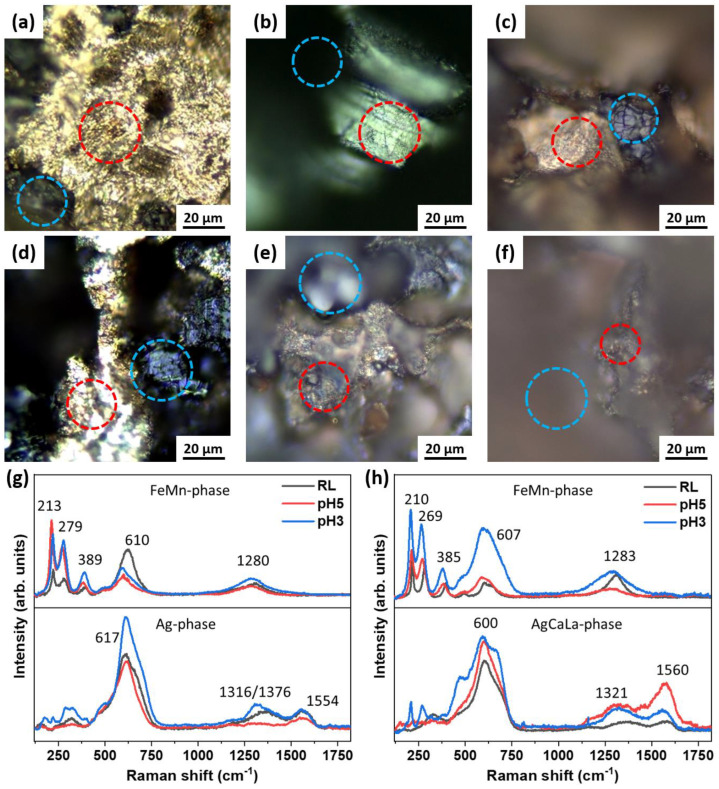
Samples after degradation (37 °C, aeration): Light microscopy image of FeMnAg: (**a**) 1244 h, RL, (**b**) 312 h, pH5, (**c**) 192 h, pH3; Light microscopy image of FeMnAgCaLa: (**d**) 1244 h, RL, (**e**) 240 h, pH5, (**f**) 144 h, pH3; (**g**,**h**) Raman spectra of the Ag and AgCaLa phase (red circles) and the FeMn phase (blue circles) at different pH values.

The Raman spectra of samples after degradation in RL, pH5, and pH3 electrolytes are shown in Figure 5. The Raman spectra were assigned to specific microscopically identified characteristic regions.

The Raman spectra of the FeMnAg and FeMnAgCaLa samples for the FeMn phase (blue circles in Figure 5a–f) and the Ag phase (red circles in Figure 5a–f) are shown in Figure 5g,h. For the FeMn phase, peaks were observed at 213 cm^−1^, 279 cm^−1^, 389 cm^−1^, 610 cm^−1^, and 1280 cm^−1^, which are characteristic of α-Fe_2_O_3_ [67]. The most relevant peaks in the spectra of the FeMn phases of both samples are similar for all pH conditions. In the areas of Ag and AgCaLa phases, significant broad absorption bands at around 600 cm^−1^ were observed. This peak might be assigned to Fe_3_O_4_ or Ag-O stretching and bending modes, respectively [42,68]. The band at 1300 cm^−1^ is assigned to the carboxylic acid group and can be explained by the lactic acid in the RL solution. Finally, the band at around 1550 cm^−1^ is assigned to AgCl [69]. The results of Raman spectroscopy showed that similar surface layers were formed on FeMnAg and FeMnAgCaLa alloys immersed at different pH values. However, the spectra support the assumption that the Ag and Ag-rich phases act as local cathodes during the corrosion process, as already predicted based on the SKPFM mappings.

**Figure 6:**
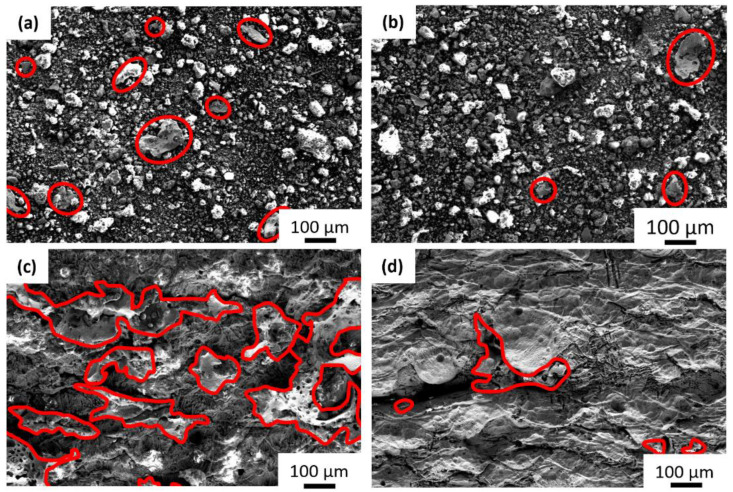
Degradation of Ag phases after 6-month immersion (37 °C, RL, aeration), SEM-SE: Particles remaining at the bottom of the immersion vessel; Ag identified via EDS and red-marked: (**a**) FeMnAg, (**b**) FeMnAgCaLa. Samples after immersion, particles marked with red border: (**c**) FeMnAg, (**d**) FeMnAgCaLa.

After immersion over six months, the Ag and AgCaLa particles released from the matrix are observed in the residues in the immersion vessel (Figure 6a,b). Significantly more particles are identified via SEM-EDS in the residues of FeMnAg (Figure 6a) than in the residues of FeMnAgCaLa (Figure 6b). Additionally, much more remaining Ag is observed on the FeMnAg sample (Figure 6c) than on the FeMnAgCaLa sample (Figure 6d). As both materials contain a similar amount of Ag and AgCaLa before immersion, the AgCaLa dissolves.

**Figure 7:**
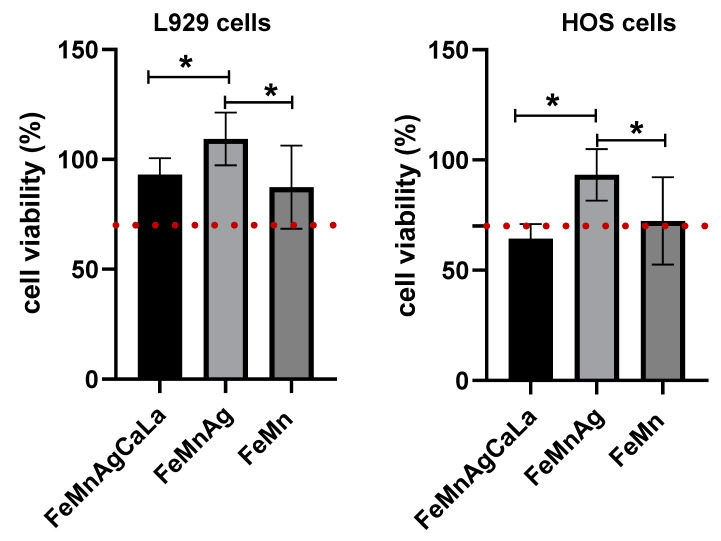
Effects of incubation with degradation media on the viability of L-929 and HOS cells after 24 h; the red dotted line demonstrates the lower limit at 70% to the reduced cell viability according to DIN ISO 10933-5; mean ± SD, n = 6 with 6 technical replicates, Kruskall–Wallis test with Dunn’s multiple comparisons test; *p* < 0.005 = * (GraphPad Prism 9.1.0.; GraphPad Software, LLC, San Diego, Cl, USA).

After 24 h in degradation media, the cell viability is affected in each case (Figure 7). Reduction below the threshold of 70% (DIN ISO 10993-5) is found in the HOS cells for viability in the case of FeMnAgCaLa. A similar reduction is demonstrated for FeMn samples. Significant differences can be found between FeMnAg and FeMnAgCaLa and FeMn, respectively.

**Figure 8:**
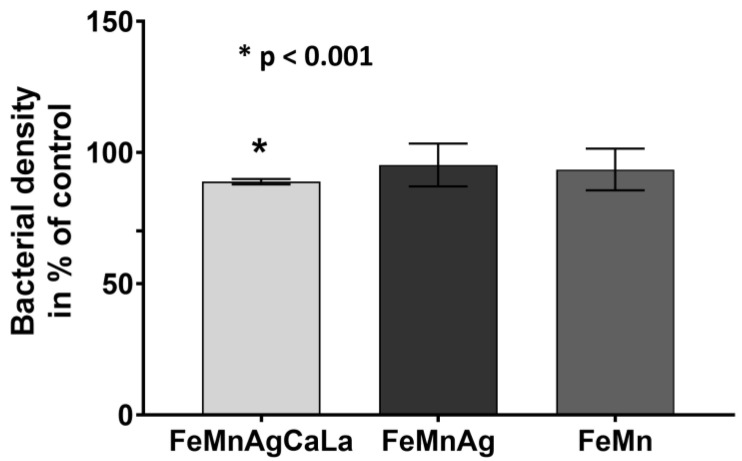
Effect of degradation supernatant on the growth of the reference strain *Escherichia coli* (ATCC^®^ 25922). Results represent effects caused by the previous degradation in 3 mL Mueller–Hinton broth. Mean value +/− standard deviation from four replicates. The optical density of the untreated control was set as 100%. Repeated measures analysis of variance (ANOVA) summary with Holm–Šídák’s multiple comparisons test in comparison to control; *p* < 0.001 = * (GraphPad Prism 9.1.0.; GraphPad Software, LLC, San Diego, CL, USA).

The degradation media of FeMnAgCaLa led to a significant reduction in bacterial growth compared to the reference, while FeMn and FeMnAg induced only a slight reduction in bacterial growth (Figure 8).

## 4. Discussion

The results prove the potential of FeMn modified with AgCaLa as resorbable material, since the degradation of AgCaLa after the dissolution of the FeMn matrix and biocompatibility are observed. Nevertheless, the cell viability is reduced below the threshold of 70% (DIN ISO 10993-5) in HOS cells treated with AgCaLa. However, this result is negligible, as FeMn and FeMnAg affect the cell viability as well, and FeMn is biocompatible (cf. introduction) [13,19,27,28,29,30,31]. As intended, an antibacterial effect is caused by FeMnAgCaLa, probably due to released Ag ions forming the degradable AgCaLa. Additionally, the fundamental proof of concept is successful, since the addition of Ag and AgCaLa enhances the degradation rate at low pH values, and small gaps surrounding Ag and AgCaLa are observed at neutral pH values. Some challenges remain, since the degradation rate is not increased at neutral pH values. The ineffectiveness of Ag and AgCaLa is confirmed by the similarity of LSV and the identical higher OCP of the modified FeMn, indicating similar and/or lower degradation rates. An in-depth analysis of the degradation behavior is necessary to achieve an understanding of the degradation mechanisms and enable the identification of the effect(s) leading to a lower anodic dissolution than theoretically possible. Based on this, adaptions of the material can be implemented.

To explain the ineffectiveness of Ag, the absence of a potential difference between Ag phases and the matrix would be a possible explanation, since the pure Ag takes up Mn from the matrix during LBM, and AgCaLa is affected by the inclusion of slag [63,64]. The SKPFM proves the higher potential of the Ag and AgCaLa phases compared to the matrix. In addition, the higher resistance of Ag and AgCaLa against corrosion is confirmed by the remaining Ag and AgCaLa, while the FeMn matrix dissolves. Thus, the absence of a potential difference is not reasonable for the ineffectiveness of the Ag and AgCaLa.

A further explanation might be the formation of corrosion-inhibiting layers due to the deposition of corrosion products. This hypothesis is supported by the depositions observed on the samples. The detection of O and, in particular, P and Cl via EDS on the Ag and AgCaLa phases is a hint of the presence of heavily dissolvable Ca-P and Ag-Cl compounds, suppressing further degradation [13,14,19,30,36,70]. Tonna et al. [14] reported a reduced degradation rate for Hank`s solution containing Ca in contrast to Hank`s solution containing no Ca. Thus, the degradation removal is reduced in case of possible Ca-P formation. This is following the results presented in this study. However, for the presented results, the formation of Ca-P is limited by the absence of P in NC and RL and not the absence of Ca. The Raman spectra confirm the presence of Ag-Cl on Ag and AgCaLa. The hypothesis of inhibiting depositions is supported by the formation of gaps around the Ag and AgCaLa phases. No depositions exist at the beginning of degradation, and the potential difference can effectively increase the dissolution of the matrix (FeMn), resulting in gaps [71]. During the development of the gaps, the inhibiting layer develops, and further anodic dissolution does not occur. Gaps around Ag phases were observed by Dargusch et al. [35] in combination with an increased degradation rate. For the presented results, the influence of the gaps is too small, and the weight of the remaining Ag phases counteracts an effective increase in degradation. Furthermore, the effectiveness of Ag and AgCaLa at low pH values is in accordance with the blocking effect, since the solubility of degradation products increases at low pH values, and the absence of inhibiting layers at low pH values is conclusive [44,72,73].

However, the ineffectiveness of Ag and AgCaLa for neutral pH values is unexpected, since an increasing effect due to anodic dissolution should occur and is reported in the literature [22,31,35,39,42,74]. An increased degradation removal after 7 days of immersion in 0.9 % NaCl solution of additively processed FeMn with 5 wt.-% Ag almost identical to the material investigated in this study was reported by Niendorf et al. [22]. The Ag phases should suppress the formation of degradation-inhibiting layers [44]. However, for the presented results, this effect does not occur, since Ca, P, and Ag-Cl are identified on FeMn and Ag, indicating the deposition of inhibiting layers. Wang et al. [44] assumed that the cathodic reaction releasing hydroxyl ions might support the deposition on the Ag phases. This hypothesis follows the reported ineffectiveness of phases with high potential [29,75,76,77]. Loffredo et al. [75] observed a slight reduction in degradation removal after immersion in Hank`s modified solution for 14 days due to modification with Ag. They suggest that the earlier formation of inhibiting layers is the reason.

Thus, the results and explanations for the degradation behavior reported in the literature differ, and the obtained results are in accordance with some studies [78]. The differences between the studies are crucial to explaining the discrepancy of degradation and identifying the decisive influencing properties in order to adapt them. As the presented results demonstrate, the immersion conditions are more decisive than the chemical composition of the materials. The results of Pierson et al. [33] underline this relationship, since material implanted into the arterial wall degrades, whereas no corrosion occurs in the bloodstream for the same material. The local differing degradation, which is also reported in the literature, emphasizes the sensitivity to immersion conditions, since the differences are caused by the local variation of conditions over the sample surface [36,79]. Accordingly, the influence of the immersion conditions is considered below.

A significant difference between the degradation in NC, RL, and HS is present. HS is the only solution containing P, and, thus, Ca-P compounds may form layers that significantly affect further degradation [13,19,28,30,36,45,71]. Therefore, the degradation in HS might be suppressed by the deposition of compounds originating from the solution. The reproduction of the significant mass removal in NC and RL, which might be suitable for some applications, is not expectable in vivo due to the presence of P, but the presence of P is not suitable as the only explanation for the ineffectiveness, since, in the literature, degradation is reported for immersion in P- and Ca-containing solutions and in in vivo experiments with the presence of Ca and P [12,13,19,35,38,39,65,79,80]. Accordingly, further aspects have to be considered. Dryndra et al. [28], Kraus et al. [29], and Peuster et al. [18] reported no significant degradation in the absence of significant inflammation, whereas Mandal et al. [38], Feng et al. [32], and Dargusch et al. [35] reported degradation in combination with an inflammatory response. These results are conclusive, since the pH value is reduced when an inflammatory response occurs, and a reduced pH value significantly affects the degradation rate, as the results presented in this study demonstrate [44,72,81]. The inflammatory response might explain the different results of in vivo studies. Otherwise, Lin et al. [16] report degradation together with slight inflammation. Thus, no single decisive parameter is identifiable for the ineffectiveness of Ag and AgCaLa. The degradation behavior results from a complex interaction of all characteristics of immersion conditions such as the pH value, the presence of P, and the material properties.

However, the results and explanations for the degradation behavior differ in the literature. Many influences are crucial, and, thus, the secure identification of reasons for the observed results is challenging. The drawn conclusions are based on suggestions about the most important influences. Even for this study, the conclusions are based on the experimental results linked with the information from the literature. Some contradictions are present, and this must be considered for the judgment of the conclusions drawn from the discussion.

In summary, the immersion conditions are crucial for the degradation behavior, and expected anodic dissolution might occur, as reported in the literature, under specific immersion conditions. However, for the successful application of the material, a reliable dissolution of the implant under all conditions would be favorable. The degradation rate must be suited regardless of an eventually occurring inflammatory response. It is challenging to fulfill these requirements since the results presented demonstrate the strong impact of the immersion conditions. Thus, the development of a material adapted for many possible applications is not expectable. Since the suitability of the material and the increased degradation by anodic dissolution might occur at some implantation sites, focusing on a more detailed simulation of conditions in the human body is appropriate. Although the degradation rate is not increased under neutral pH values, further investigations are needed, since the effectiveness of the concept of anodic dissolution by modification with Ag, in general, is proven. The degradation rate is increased for low pH values, and gaps develop around Ag for neutral pH values. Furthermore, an adaption of the material is appropriate to enable the application for more use cases. A possibility is to introduce more, smaller AgCaLa phases to increase the impact of the gaps around the Ag phases. In addition, the formation of Mn segregations is promising, since a significant influence of segregations is observed. However, inhibiting layers are developed. Thus, the development of an additional mechanism to prevent the deposition of such layers is required to enable the effectiveness of potential differences [71].

## 5. Conclusions

The results presented demonstrate the impact of the modification of FeMn with Ag and AgCaLa, a degradable Ag-alloy, on the biocompatibility, antibacterial properties, and degradation behavior. Furthermore, an analysis of the degradation behavior of the FeMn matrix and the Ag phases identifies crucial factors of the immersion conditions and enables further investigations and adaptions of the material. The key findings can be summarized as follows:

- For low pH values, the addition of Ag increases the degradation rate.
- For neutral pH values, small gaps surrounding the Ag and AgCaLa phases develop.
- For immersion conditions adapted to the human body, no significantly increased degradation rate occurs.
- The Ag phases effectively cause anodic dissolution, but depositions of inhibiting layers suppress an increased degradation rate for the selected immersion conditions.
- The degradation behavior strongly depends on the pH value and the composition of the immersion solution—particularly, the presence of P.
- Mn segregations influence the local degradation behavior.
- Particles of the degradable AgCaLa alloy dissolve after the matrix material.
- Biocompatibility can be assumed for all materials.
- An antibacterial effect is observed for FeMn modified with degradable AgCaLa.

In summary, the results demonstrate the possibility of increasing the degradation rate by the addition of phases with high electrochemical potential, causing anodic dissolution. The results also reveal remaining challenges, as, for the selected immersion conditions with a neutral pH value, the degradation rate is not increased. Since high sensitivity to the immersion conditions is observed, the investigated material might be effective on some implantation sites. Accordingly, further research should focus on a more detailed simulation regarding the immersion conditions. Furthermore, an adaption of the material with smaller Ag phases or applying the influence of Mn segregations should be aimed for. To enable the effectiveness of the actual material, a mechanism to suppress the formation of inhibiting layers needs to be developed.

## Figures and Tables

**Table 1:** Combination of immersion conditions and solutions.

	Condition	Aeration 20 °C	Aeration 37 °C	Bubbled Air 20 °C	Bubbled Air 37 °C
Solution					
Ringer–Lactate Solution (RL)		X	X	X	X
Hanks’ Solution (HS)		X	X	X	X
NaCl Solution (NC)		X	X	X	X
pH5			X		
pH3			X		

**Table 2:** Composition of solutions utilized for static immersion tests.

Components		Concentration g/L				
		HS	RL	NC	pH5	pH3
Calcium Chloride Dihydrate	CaCl_2_ • 2H_2_O	0.186	0.270	---	---	---
Dextrose	C_6_H_12_O_6_	1.000	---	---	---	---
Magnesium Sulfate Heptahydrate	MgSO_4_ • 7H_2_O	0.200	---	---	---	---
Potassium Chloride	KCl	0.400	0.400	---	---	---
Potassium Phosphate Monobasic Anhydrous	KH_2_PO_4_	6.000×10^−2^	---	---	---	---
Sodium Bicarbonate	NaHCO_3_	0.350	---	---	---	---
Sodium Chloride	NaCl	8.000	6.000	9.000	---	1.690
Sodium Phosphate Dibasic-7-Hydrate	Na_2_HPO_4_ • 7H_2_O	9.000×10^−2^	---	---	---	---
Phenol Red	C_19_H_14_O_5_S	2.000×10^−2^	---	---	---	---
Sodium Lactate	NaC_3_H_5_O_3_	---	3.120	---	---	---
Citric Acid	C_6_H_8_O_7_	---	---	---	0.940	3.760
Caustic Soda	NaOH	---	---	---	0.400	0.400

## Data Availability

Not applicable.
